# Physico-Chemical Characteristics and In Vitro Gastro-Small Intestinal Digestion of New Zealand Ryegrass Proteins

**DOI:** 10.3390/foods10020331

**Published:** 2021-02-04

**Authors:** Lovedeep Kaur, Harmandeepsingh Lamsar, Ignacio F. López, Manon Filippi, Dayna Ong Shu Min, Kévin Ah-Sing, Jaspreet Singh

**Affiliations:** 1 School of Food and Advanced Technology, Massey University, Palmerston North 4442, New Zealand; L.kaur@massey.ac.nz (L.K.); hrmn87nz@gmail.com (H.L.); manon.filippi@gmail.com (M.F.); 1601298@sit.singaporetech.edu.sg (D.O.S.M.); kevin.ahsing@gmail.com (K.A.-S.); 2 Riddet Institute, Massey University, Palmerston North 4442, New Zealand; 3 School of Agriculture and Environment, Massey University, Palmerston North 4442, New Zealand; I.F.Lopez@massey.ac.nz

**Keywords:** grass protein concentrate, solubility, in vitro digestion, thermal denaturation

## Abstract

Being widely abundant, grass proteins could be a novel source of plant proteins for human foods. In this study, ryegrass proteins extracted using two different approaches-chemical and enzymatic extraction, were characterised for their physico-chemical and in vitro digestion properties. A New Zealand perennial ryegrass cultivar *Trojan* was chosen based on its higher protein and lower dry matter contents. Grass protein concentrate (GPC) with protein contents of approximately 55 and 44% were prepared using the chemical and enzymatic approach, respectively. The thermal denaturation temperature of the GPC extracted via acid precipitation and enzymatic treatment was found to be 68.0 ± 0.05 °C and 66.15 ± 0.03 °C, respectively, showing significant differences in protein’s thermal profile according to the method of extraction. The solubility of the GPC was highly variable, depending on the temperature, pH and salt concentration of the dispersion. The solubility of the GPC extracted via enzymatic extraction was significantly lower than the proteins extracted via the chemical method. Digestion of raw GPC was also studied via a gastro-small intestinal in vitro digestion model and was found to be significantly lower, in terms of free amino N release, for the GPC prepared through acid precipitation. These results suggest that the physico-chemical and digestion characteristics of grass proteins are affected by the extraction method employed to extract the proteins. This implies that selection of an appropriate extraction method is of utmost importance for achieving optimum protein functionality during its use for food applications.

## 1. Introduction

Plants have been used in the human diet as sources of energy and nutrients for many years. Plants protein market is growing rapidly as plants are seen as a sustainable solution to meet nutritional needs of the growing world population [1]. Perennial crops such as grasses can generate a large amount of biomass and being widely abundant, could be a potential source of protein for human consumption. The enzyme, 1,5-bisphosphate carboxylase oxygenase (RuBisCO), which is a chloroplastic protein, makes up approximately 50% of the protein extracted from the leaf [2,3]. Other proteins present in green leaves are lipoproteins, structural proteins, pigment bound proteins and enzymes [2].

As RuBisCO is the main protein component in grass, it determines the functionality and nutritional value of grass protein products. It has been reported to possess an excellent complement of essential amino acids and is very rich in tryptophan, leucine, tyrosine and phenylalanine compared with other food proteins and the FAO/WHO reference pattern [2]. RuBisCO has also been reported to possess desirable functional properties such as gelation, foaming, and emulsification which might enable food processors to successfully incorporate the protein into a number of different food systems [2]. However, extraction processes greatly influence the solubility, denaturation state and composition of protein isolates. This in turn influence their functional properties such as gelation, water binding, emulsifying and foaming capacities, which are relevant for food applications [4]. Moreover, the plant species from which the protein is extracted play a significant part in affecting results [5]. The solubility of RuBisCO extracted via acid precipitation from alfalfa, soybean leaf, sugar beet leaf and tobacco varied greatly, depending on the solvent used, pH, temperature, ionic strength, and protein concentration [6]. The use of antioxidants such as sodium sulphite during protein extraction has been reported to increase the soluble RuBisCO content in red clover possibly by inactivation of polyphenol oxidase enzyme thereby preventing cross-linking between polyphenols and soluble RuBisCO [7].

A range of conventional solvent based protein extraction methods are predominantly being used in the food sector. Both dry and wet protein extraction techniques require cell disruption as initial phase to allow protein release from protein bodies inside plant cells. Classically, cell disruption is performed by mechanical methods (e.g., grinding, milling) or by thermal and chemical treatments. However, due to high sensitivity of proteins to heat or solvent use, novel processing technologies have emerged and used for cell disruption demonstrating more efficient yield, extraction time, costs and environmental impact [8]. Some of the most promising techniques are ultrasound- and enzyme-assisted extraction, microwave-assisted extraction, pulsed electric field assisted extraction, supercritical fluid extraction and pressurised liquid extraction [9,10].

Enzyme assisted extraction of biomolecules from plants has been considered to be a potential alternative to conventional solvent extraction methods and is gaining more attention because of being an efficient, benign, sustainable and eco-friendly extraction technology [11]. Routine extraction processes are not able to access the components which are dispersed in the cell cytoplasm of plant matrices and retained in the polysaccharide-lignin network by hydrogen or hydrophobic bonding [12] The addition of enzymes like cellulase, hemicellulase, pectinase and α-amylase during extraction enhances recovery by the cell wall breakdown and hydrolysing the structural polysaccharides and lipid bodies [12]. Various factors including enzyme composition and concentration, particle size of plant materials, solid to water ratio, and hydrolysis time are recognised as key factors for extraction [9]. Enzyme-assisted protein extraction is associated with irreversible carbohydrate-protein matrix disruption and requires adjustment of process parameters such as pH and temperature; but involves long processing time, high operational costs and high energy consumption [8]. However, it has lower environmental impact and considered a milder extraction method when compared to acid and alkaline assisted extraction methods [13]. Moreover, products obtained from enzyme-assisted extraction are considered to be of superior and preserved quality and more suitable for human consumption [8].

The aim of the present study was to compare the physico-chemical properties and in vitro digestibility of grass protein concentrate (GPC) extracted via acid precipitation and enzyme assisted extraction. The *Trojan* cultivar, a perennial ryegrass (*Lolium perenne* L.) which was released in New Zealand in 2005, was chosen. This perennial ryegrass comes from a re-selection from the cultivar *Tolosa* which delivered excellent persistence. *Trojan* has a better protein content (24% protein dry basis) and provide a higher total dry matter yield by producing 9% more than standard ryegrass across all seasons [14].

There is no regulatory status for a grass protein concentrate. However, the Codex Alimentarius Commission (Codex general standard 174-1989; joint FAP/WHO Food standards Programme) [15] on Vegetable Products (CCVP) has developed an international general standard for vegetable protein products (VPP). The VPP are defined as “food products produced by the reduction or removal from vegetable materials of certain of the major non-protein constituents (water, oil, starch, other carbohydrates) in a manner to achieve a protein content of 40% (dry weight basis) or over” [15].

## 2. Materials and Methods

### 2.1. Raw Materials and Chemicals

The *Trojan* cultivar of perennial ryegrass, developed in New Zealand, was used for the experiments. The enzyme used for the extraction was Viscozyme L, a multi-enzyme complex from *Aspergillus* spp. containing carbohydrases including arabanase, cellulase, β-glucanase, hemicellulase and xylanase (V2010-250mL, Sigma Aldrich, Schnelldorf, Bavaria, Germany) and stored at 5 °C. This enzyme complex is capable of hydrolysing structural cell wall polysaccharides, improving extraction and obtaining protein concentrate [16]. It complies with the recommended purity specifications for food-grade enzymes given by the joint FAO/WHO Expert Committee on Food Additives (JECFA) and the Food Chemical Codex (FCC). All chemicals used in the study were of analytical grade.

### 2.2. Preparation of Grass Protein Concentrate Using Acid Precipitation

*Trojan* ryegrass was harvested and frozen at −20 °C until further required. GPC was extracted and precipitated using a method described by Zhang, et al. [17] with some modifications. The frozen grass was mixed with distilled water (58.8% *w*/*v*) and 0.1 M NaOH (26% *v*/*w*). The mixture was then ground using a wet disintegrator (C200, JEFFRESS Engineering Pty Ltd., Dry Creek, Australia) at 6000 rpm for 10 min. The mixture was pressed through a cheese press (Massey University, Palmerston North, New Zealand) and the filtrate was collected. The process was then repeated with the residue obtained from the filtration process, and the filtrate was centrifuged (Westfalia CTC-03-107). Precipitation was then carried out at pH 3.5 and centrifuged again to collect the protein precipitate. The protein precipitate was freeze-dried, vacuum packaged and stored at 4 °C until analysed.

### 2.3. Preparation of Grass Protein Concentrate Using Enzyme Treatment

**Figure 1:**
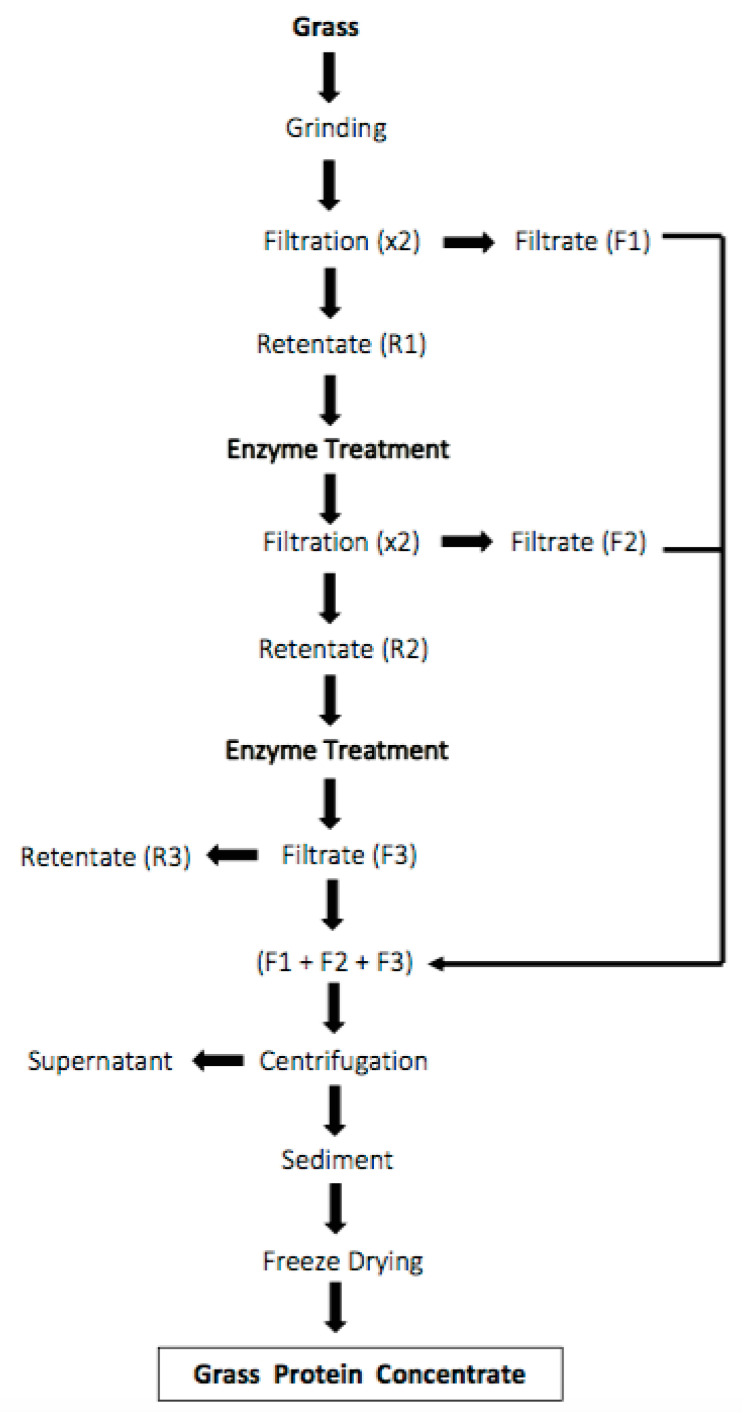
Process flow chart for protein extraction by enzyme treatment.

Based on preliminary work, a new extraction process stated in Figure 1 was standardised for extraction of GPC using the enzyme treatment. The frozen grass was ground with 1.5 times of water using a wet disintegrator (C200, JEFFRESS Engineering Pty Ltd., Dry Creek, Australia) for 10 min. Then the grass slurry was put in a Cheese Press (Massey University, Palmerston North, New Zealand) to remove all of the solids part. The process was repeated with the residue obtained from the filtration process by adding additional water (9000 mL).

The solids parts obtained (R1) were subjected to enzyme treatment for 3 h. This treatment was performed using the multi-enzyme complex Viscozyme L at 1% concentration. The enzyme solution to wet grass ratio was 7:1 (*v*/*w*), while the pH and temperature were maintained as 5 and 45 °C, respectively. These conditions were chosen based on preliminary experimentation to get better protein yield. The temperature of the mixture was maintained using an Electric Jacketed Pan and the pH was adjusted using 6 M HCl. After 3 h of treatment, the mixture was put in the industrial press again, ground with water (10 L) and pressed through a cheese press again.

Then the retentate/solids obtained (R2) were subjected to a second enzyme treatment under the same operating conditions as the first treatment. After further 3 h of treatment, the mixture was put in the industrial press, re-ground with water (5 L) and pressed through a cheese press again.

All the filtrates obtained (F1, F2 and F3; Figure 1) were collected, mixed and centrifuged using a Westfalia Separator CTC-03-107 (GEA, Oelde, Germany). The sediment obtained was frozen and freeze-dried (FD18LT Freeze Drier, Cuddon, Blenheim, New Zealand). Then the powder was ground in a coffee grinder (BCG200, Breville^®^, Sydney, Australia), vacuum packaged and stored at −4 °C until analysed.

### 2.4. Proximate Analysis

The moisture content was determined using convection oven at 105 °C (AOAC 930.15925.10) [18] and calculated using the weight difference between the samples before and after drying. The protein content of samples was determined using the Kjeldahl method (AOAC 978.04) [18] and the nitrogen conversion factor of 5.83 was applied [19].

GPC prepared through acid precipitation was also subjected to nutritional analysis, including ash; fat; total, soluble and insoluble dietary fibre; carbohydrate; and minerals (calcium, potassium, magnesium, sodium and iron) using AOAC 942.05; AOAC 2003.06; Megazyme AOAC 991.45 [18]; starch + sugars method; and inductively coupled plasma-optical emission spectroscopy (ICP-OES) [20] methods, respectively.

### 2.5. Differential Scanning Calorimetry

The methodology for the DSC analysis was adapted from Ahmad, et al. [21], with slight modifications. Twelve (12 mg) milligrams of GPC was weighed into aluminium pans (TA Instruments, TZero 901684.901), followed by the addition of 18 mg of Milli-Q water to obtain 30 mg of 40% *w*/*v* GPC concentration. The pans were then hermetically sealed and left overnight at 23 ± 2 °C. Scans were then conducted using a differential scanning calorimeter (Q2000, TA Instruments, New Castle, DE, USA) in triplicates, with heating from 20 to 100 °C at a rate of 5 °C/min. The DSC was calibrated with indium and gallium, with empty aluminium pans used as reference. The thermal properties of the GPC were then analysed using an analytical software (TA Universal Analysis, TA Instruments, New Castle, DE, USA). The thermal denaturation temperature (T_d_) was determined based on the highest peak of the endothermic curve, and the enthalpy was calculated from the area below the transition peak.

### 2.6. Effect of pH and Temperature on Solubility of Grass Protein Concentrate

Solubility of the protein powder was determined using the methods from Bolontrade, et al. [22], with slight modifications. In short, GPC was first dissolved (1%, *w*/*v*) in distilled water. To determine the effect of pH on solubility, the pH of the solution was then adjusted using 1M HCl or NaOH to pH 1.5–9 (pH 7, 9, 10 and 12 for GPC prepared through enzyme treatment). For effect of salt (NaCl) concentration, GPC (1%, *w*/*v*) was dissolved in 0.1–1 M NaCl solutions with the pH adjusted to 9. The solutions were then mixed at 300 rpm for 1 h at 20 °C.

For studying the effect of temperature, GPC was dissolved in distilled water (1%, *w*/*v*) with the pH adjusted to 7 and 9 (pH 9 only tested for GPC prepared via enzyme treatment), and left to stir at 300 rpm for 1 h using a magnetic stirrer, at 20 °C, 40 °C, 60 °C, 80 °C or 100 °C [23]. To prevent from moisture loss during heating, the beakers were covered with aluminium foil. The pH was adjusted again after 30 min of heating treatment. The pH of the solution decreased by 2 pH units along the heating that could be due to a conformational change of the protein induced by thermal denaturation [24]. All samples were then centrifuged at 2400× *g* for 15 min, and the supernatant was tested for soluble protein concentration (Ps) using the Kjeldahl method, AOAC 978.04 [18]. Results were expressed as percentage of solubility, %S:

(1) $$Solubility\;\left(\%\right)=\frac{Ps}{Initial\;protein\;concentration}\;\times \;100$$

A pH-shifting treatment was applied to GPC solution prepared by enzyme treatment (1%, *w*/*v*) by following the method proposed by Yildiz [25] with slight modification. GPC solution was adjusted to pH 10 and 12 with 1M NaOH. The solutions were mixed at 300 rpm for 1 h at 20 °C before adjusting the pH back to pH 7 with 6 M HCl. Then the effect of temperature on GPC solution treated by pH-shifting at pH 12 was determined on protein solubility as described in above.

### 2.7. Determination of Gastro-Small Intestinal In Vitro Protein Digestibility

The GPC dispersions (1:5 *w*/*v* in water) prepared via acid precipitation and enzyme treatment tested for their in vitro protein digestibility.

#### 2.7.1. In Vitro Gastro-Small Intestinal Digestion

In vitro oral-gastro-small intestinal digestion was carried out on GPC dispersions using the method described by Chian, et al. [26]. All digestions were conducted at 37 ± 1 °C. Samples were drawn at 0, 30 and 60 min of gastric digestion and 10 μL of Pepstatin A (AB141416, Abcam Plc, New Zealand) was then added to every mL of digest collected.

For the small-intestinal digestion phase, digests were collected after 60 and 120 min of small-intestinal digestion. Protease inhibitor cocktail solution (SigmaFast^TM^, Sigma Aldrich, Schnelldorf, Bavaria, Germany) (350 μL/mL digest) was then added to the digests collected. All digests were then stored at −20 °C until further analysis.

#### 2.7.2. Soluble N and Free Amino N Contents

The digests were centrifuged at 17,000× *g* for 10 min at 4 °C using a high-speed centrifuge (CR 22 GII, Himac, Hitachi Koki Co., Ltd., Ibaraki, Japan). The supernatant was then filtered through a 0.45 µm PVDF filter. The supernatants were analysed for soluble protein (%) and free amino nitrogen using the Kjeldahl method (as described in Section 2.4) and a colorimetric method to determine the ninhydrin-reactive amino nitrogen content, as described by Moore [27] and Kaur, et al. [28], respectively.

Results are presented as means of at least triplicate measurements ± SD.

## 3. Results & Discussion

### 3.1. Protein Extraction Processes, GPC Yield (%) and Composition

The average yield of GPC powder obtained through acid precipitation was approximately 2.22% (fresh grass weight basis) and the protein content was 54.69%.

We assumed that through enzyme hydrolysis via Viscozyme, structural cell wall components would be hydrolysed, releasing intracellular components such as chloroplasts containing RuBisCO. This could be the reason for the observed higher yield of GPC obtained when mechanical process was combined with enzyme treatment compared with mechanical disintegration alone (data not shown).

The optimum pH and temperature conditions for the activity of this multi-enzyme complex have been suggested to range between 3.3–5.5 and 40–50 °C, respectively (Sigma Aldrich, Schnelldorf, Bavaria, Germany). The enzyme solution concentration used (1%) was chosen based on literature [16,29] and preliminary tests. The enzyme solution was added to the retentate at a ratio of 7:1 (*v*/*w*) to obtain a mixture in enough liquid state to ensure continuous agitation by the overhead stirrer throughout the treatment.

Preliminary work by our research group has shown that the enzyme treatment did not improve protein extraction yield when the process lasted for less than 6 h. Thus, a 6 h (3 + 3 h) treatment was chosen. The enzymatic treatment resulted in about 85% of grass proteins to be recovered. Viscozyme L might not be able to break some of the complex bonds between pectin and proteins [30] and thus prolonged enzyme treatment for 10 h showed no further effect on protein recovery (data not shown).

**Figure 2:**
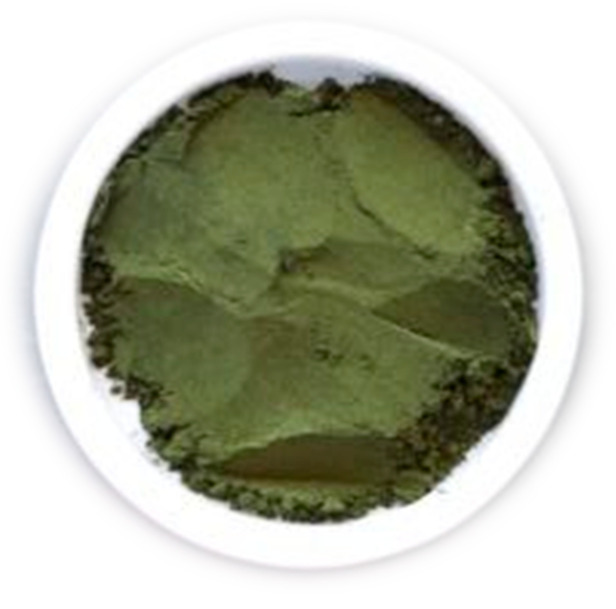
Grass Protein Concentrate prepared via enzyme extraction.

The protein content of the GPC obtained through the enzymatic process after freeze drying was 44% for the 6 h treatment. This indicated the presence of non-proteinaceous components in the GPC. The average yield of GPC obtained from this process was 11% (dry grass weight basis). The final powder obtained after grinding was homogeneous and green in colour as shown in (Figure 2).

The GPC prepared by the acid precipitation method showed its protein and fat contents quite similar to a commercially available green-coloured Spirulina powder [31] while the sodium content was about six times lower than the latter (Table 1). It is also a significant source of other minerals including, calcium, potassium, magnesium and iron, suggesting the suitability of GPC as a dietary supplement.

### 3.2. Thermal Stability of the Grass Protein Concentrate

When a protein is subjected to heat, it undergoes endothermic processes such as conformational changes and disruption of bonds, which unfolds and denatures the protein. Simultaneously, as heat is applied, forces such as hydrogen bonds and van der Waals forces that holds the protein’s tertiary and quaternary structures are weakened. This exposes buried hydrophobic sites [32], causing aggregation of the protein molecules, which is an exothermic process. The differential scanning calorimeter (DSC) hence measures the net changes between the endo- and exothermic processes [33].

Raw GPC samples extracted through acid precipitation and enzymatic treatment were subjected to DSC analysis. Results obtained showed that the T_d_ was 68.0 ± 0.05 °C and 66.15 ± 0.03 °C for samples prepared through acid precipitation and enzymatic treatment, respectively, which indicated that the GPCs extracted via different methods undergo conformational changes and unfold at different temperatures. This is in agreement with the studies conducted on RuBisCO obtained from other sources, such as alfalfa and tobacco. The protein RuBisCO from alfalfa displayed a T_d_ of 61.85 to 72.85 °C [34,35], depending on the experimental conditions utilised during the DSC measurements.

The enthalpies of denaturation of the GPC obtained via acid precipitation and enzyme treatment were determined to be 2.26 ± 0.22 J/g and 3.52 ± 0.39 J/g, respectively. These values differ from the reported value of 26.3 J/g from RuBisCO extracted from alfalfa [36]. This was speculated to be due to the presence of proteins other than RuBisCO and also to differences in pH of the GPC suspensions in the current study. Béghin, Bizot, Audebrand, Lefebvre, Libouga and Douillard [34] stated that the enthalpy of alfalfa RuBisCo decreased at pH below 7. This was attributed to a minimisation of intramolecular electrostatic repulsions near the isoelectric point. A lowering of protein-protein repulsion forces might have caused an irreversible aggregation which resulted in the observed differences in the thermal properties [37].

### 3.3. Solubility of GPC

Proteins that have high solubility allows for a uniform dispersion of protein particles to be obtained to form a good colloidal system. Proteins with high solubility have an increased number of potential applications in foods [38]. Hence, to determine the ability of the GPC to be utilised in food applications, it was solubilised under varying conditions, and the total soluble nitrogen content was plotted against the pH, temperature and salt concentrations of the solutions.

#### 3.3.1. Effect of pH on Protein Solubility

**Figure 3:**
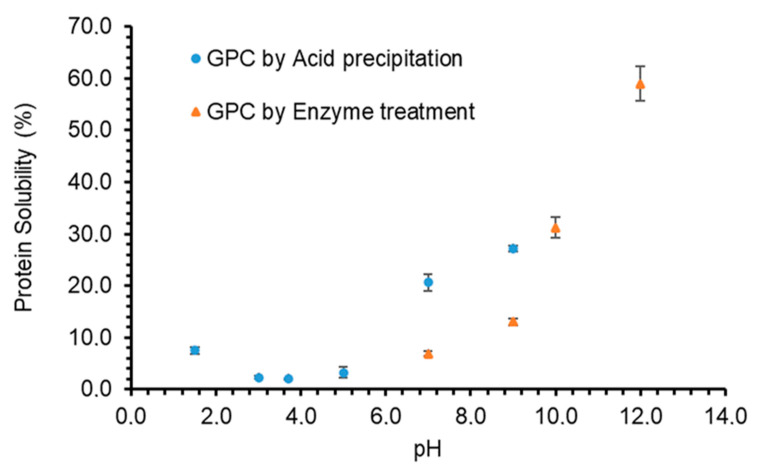
The effects of pH on protein solubility (%) of grass protein concentrate (GPC) extracted through acid precipitation or enzyme treatment. Values are means (*n* = 3) ± SD.

When the pH of the GPC solution was varied, a significant effect on protein solubility was observed (*p* < 0.05), which indicates that the solubility is highly dependent on the pH of the environment (Figure 3). The protein solubility of GPC extracted by acid precipitation method was found to be lowest in the range of pH 3–5, and it increased significantly when pH was increased up to 9. According to several studies conducted on protein solubility, it has been concluded that a protein is least soluble at pH nearest to its isoelectric point [23]. This is due to an increase in protein-protein interactions, as electrostatic forces of the protein molecules are at its lowest (no net electrical charges), leading to little water interaction with protein molecules. At this point, the proteins tend to aggregate, resulting in a decrease of protein solubility [39]. However, the minimum solubility experienced by the GPC in this study did not occur at the isoelectric point of pure RuBisCO, which is reported to be in the region of pH 4–6 [6,40]. This deviation may have occurred as the GPC sample used in this study was not a purified form of RuBisCO and may contain other forms of protein such as ATP synthase or protein kinase, which are proteins present in green leaves [41]. Similarly, the process used to obtain the GPC in this study might have differed from other studies. Different methods of obtaining RuBisCO, such as through thermal or pH denaturation, have been reported to affect its solubility [33,42,43].

When pH of the GPC solution was increased, the solubility of GPC extracted by acid precipitation was found to be 20% and 27% at pH 7 and 9, respectively. On the other hand, the solubility of GPC extracted by enzyme treatment was only 6.8% and 13.3% at pH 7 and 9, respectively. Nevertheless, GPC from enzyme treatment had better solubility in alkaline pH and it increased to 31.2% and 58.98% at pH 10 and 12, respectively at 20 °C. These results show that solubility is affected by the method of extraction. It has been reported that proteins obtained from leaf sources via various preparation methods have displayed a similar trend of having a higher protein solubility on either side of the isoelectric point [33,42,44]. Protein solubility of grass pea protein concentrate also reportedly decreased with a decrease in pH until pH 4–5 followed by an increase with an increase in pH [4]. This is also observed in the solubility curve of the GPC, which is due to proteins acquiring net positive or negative charges when in acidic and basic environments. In alkaline environment, proteins acquire a total negative charge on their surfaces, and electrostatic repulsions between the molecules increase, decreasing protein-protein interactions and increasing the protein-water interactions, therefore the solubility [45].

**Figure 4:**
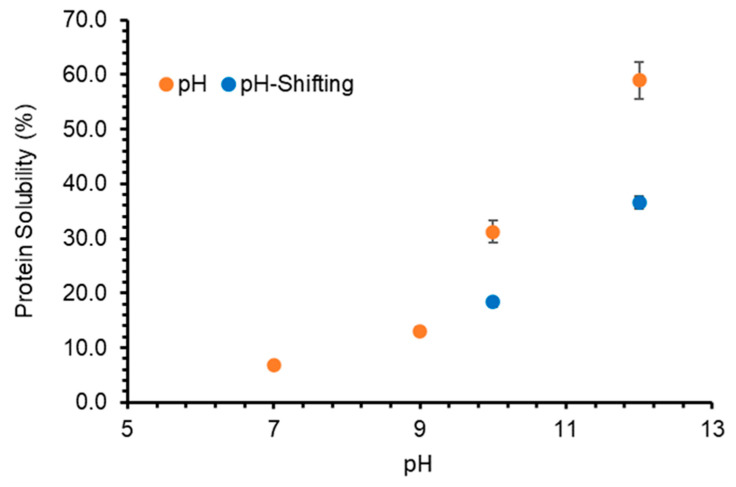
The effects of pH-shift on protein solubility (%) of grass protein concentrate (GPC) extracted through enzyme treatment. Values are means (*n* = 3) ± SD.

To further improve the solubility of enzyme-extracted GPC at neutral pH, the effect of the pH-shifting process was studied. The pH-shifting process involved subjecting the protein solution to an extreme acidic or alkaline pH condition and then to a neutral pH environment that allows the protein to undergo partial unfolding and then refolding to assume a molten globule conformation that has unique surface properties [46]. The solubility of the GPC extracted by enzyme treatment increased to 18.34% and 36.56% at neutral pH when the GPC was treated at pH 10 and 12, respectively at 20 °C (Figure 4). This unfolding-refolding phenomenon resulted in 5.3 times higher protein solubility at neutral pH for pH 12-treated samples as compared to samples at neutral pH without any pH-shifting treatment. The unfolding-refolding phenomenon was described as an effective step in the modification of protein characteristics [46]. However, the structural changes appeared to primarily involve tertiary structure and could not be completely reversed with refolding treatment at pH 7, thus producing a “molten globule” structure [47].

#### 3.3.2. Effect of Temperature on Protein Solubility

**Figure 5:**
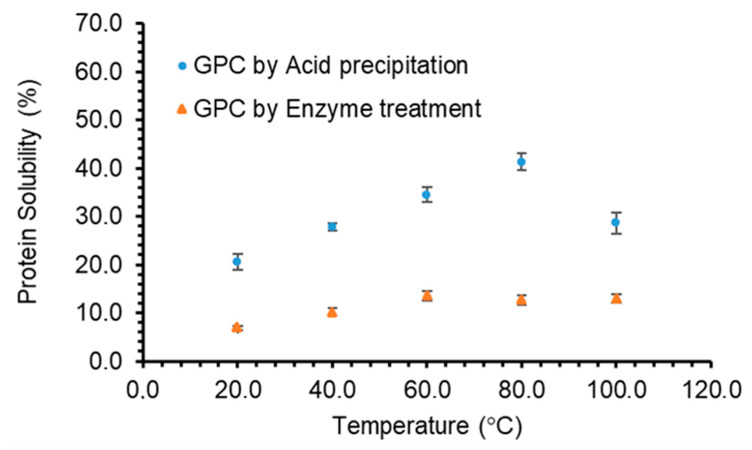
The effects of temperature on protein solubility (%) of grass protein concentrate (GPC, pH 7) extracted through acid precipitation or enzyme treatment. Values are means (*n* = 3) ± SD.

Protein solubility is highly dependent on protein-water interactions, which may be affected by the temperature of the medium the protein is subjected to. When GPC was subjected to a range of temperatures at neutral pH, the solubility increased with increasing the temperature (Figure 5) indicating more protein-water interactions resulting in an increase in the solubility. Protein denaturation during thermal treatment have also been reported to result in weakening of hydrogen bonds and electrostatic forces that hold the protein’s secondary, tertiary or quaternary structure together [23], which could also increase solubility. However, the solubility of GPC obtained by acid precipitation was found to decrease at temperatures higher than 80 °C.

**Figure 6:**
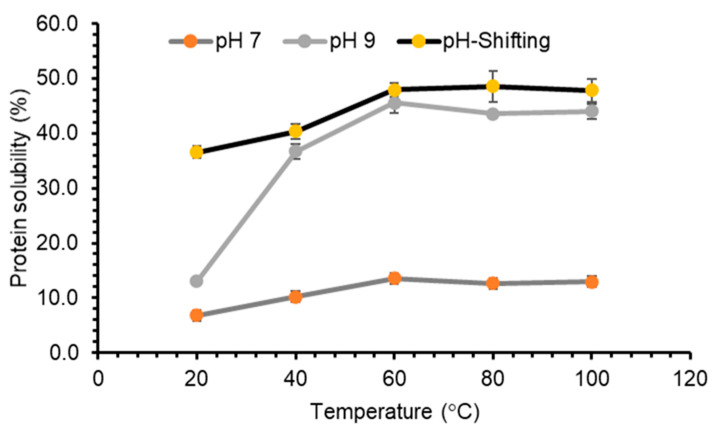
The effects of pH-shift on protein solubility (%) at different temperatures of grass protein concentrate (GPC) extracted through enzyme treatment. Values are means (*n* = 3) ± SD.

Similar results were observed for GPC extracted by enzyme treatment, where the solubility increased with an increase in the temperature up to 60 °C, at pH 7 and 9 (Figure 6). However, when GPC was subjected to temperatures above 60 °C the solubility remained stable, around 13% for pH 7 and 44% for pH 9. Likewise, when the temperature of the GPC sample, obtained by enzyme treatment treated with pH-shifting process of pH 12, was increased from 20 °C to 60 °C, the solubility increased from 36.56% to 48%, and remained stabled above 60 °C (Figure 6). This could be explained by the surface properties of proteins. The amount and distribution of hydrophilic and hydrophobic amino acid moieties on the surface may vary from one protein to another, which can have an impact on the behavior of a protein in solution [23,48].

#### 3.3.3. Effect of Salt Concentration on Protein Solubility

**Figure 7:**
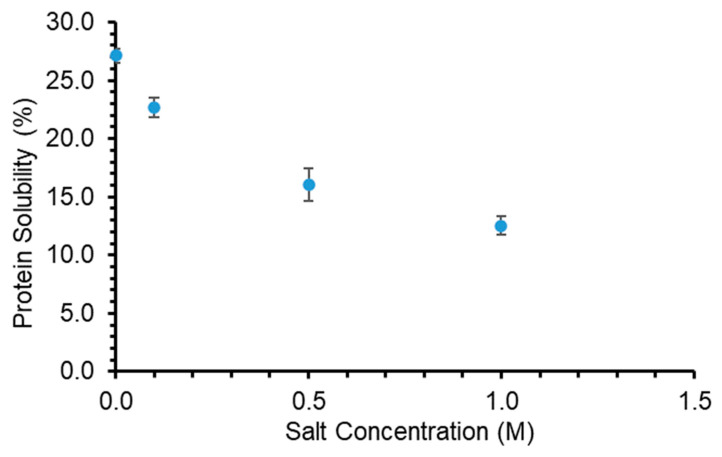
The effects of salt (NaCl) concentration (M) on protein solubility (%) of grass protein concentrate (GPC) extracted through acid precipitation. Values are means (*n* = 3) ± SD.

The solubility of protein could be affected by the ionic valence, concentration, and the type of ions dissociated by the salts added in the solution. When the concentration of sodium chloride in GPC solution was increased, it resulted in a significant decrease (*p* < 0.05) in the protein solubility as shown in (Figure 7). Salting out phenomenon was observed, whereby an increase in salt content led to a decrease in protein solubility. This occurred due to ions being dissociated into the solution, which might have reduced the electrostatic repulsive interactions between the charged protein molecules [49]. Hence, as more salt was added into the solution, more ions were dissociated which led to a greater reduction in the repulsion forces between the protein molecules, causing aggregation and precipitation of the proteins from the solution, decreasing its solubility.

### 3.4. In Vitro Protein Digestibility of GPC

#### 3.4.1. Soluble Nitrogen Content during Digestion

**Figure 8:**
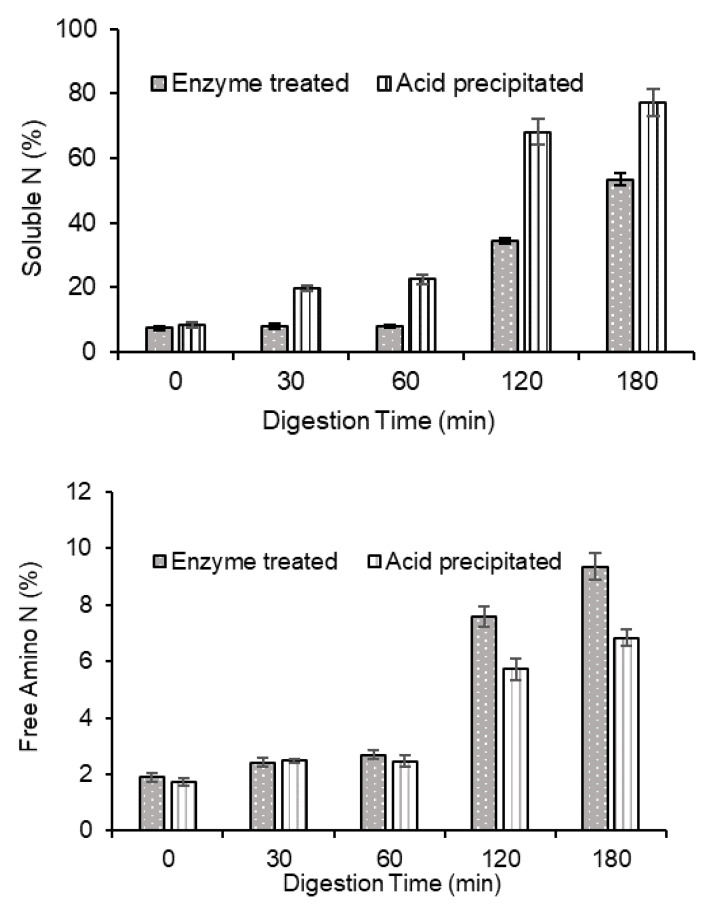
Soluble N (%, **above**) and Free amino N (%, **below**) during in vitro gastro (0, 30, 60 min)-small intestinal (120, 180 min) protein digestion for GPC extracted via acid or enzyme treatment. Values are means (*n* = 3) ± SD.

For the raw GPC prepared via either enzymatic or acid treatment, during the stomach phase of the digestion, protein solubility was poor (Figure 8). But the solubility increased significantly during the small-intestinal digestion phase. However, GPC prepared by acid precipitation had significantly (*p* < 0.05) higher solubility compared with digests from enzyme-treated GPC during both gastric and small-intestinal digestion phases.

#### 3.4.2. Free Amino Nitrogen Content

Overall, GPC displayed lower ninhydrin reactive free amino N values than those reported for muscle proteins [26,28]. High protein digestibility value has been attributed to the ease of access of the protease to the peptide bonds aided by a lower amount of non-protein materials [50]. Thus, increasing the purity of the GPC might also improve the digestibility of grass proteins. As seen in (Table 1), GPC contains a significant amount of insoluble dietary fibre (DF). Dietary fibre has been shown to affect processes that occur throughout the gastro-intestinal tract. The composition of cell walls, which is the main dietary fibre in plants, could affect the digestion process in the small intestine. Insoluble DF has been reported not to have a large impact on nutrient absorption while the presence of DF could decrease protein digestibility [51,52]. In a study conducted on rapeseed, the presence of various sources and amounts of DF reduced the in vitro protein digestibility of napin proteins, which was associated with a lowered proteolytic enzymatic activity during the in vitro protein digestion process [53]. A reduced enzyme to substrate contact was also reported to have possibly occurred due to the presence of DF [53]. Therefore, the low in vitro protein digestibility observed for GPC could be attributed to the presence of non-protein substances, particularly high insoluble dietary fibre content of GPC.

The free amino N values obtained after digestion of GPC prepared by enzyme treatment were significantly higher than those obtained for GPC prepared by acid precipitation.

## 4. Conclusions

The chemical composition of GPC (prepared by acid precipitation), particularly its protein and fat contents, was observed to be close to blue-green algae based commercial product (Spirulina), which is commonly used as a dietary supplement. GPC could also be a good source of other minerals such as calcium, potassium, magnesium and iron. The solubility of the grass protein concentrate was found to be significantly affected by the method of protein extraction, pH, temperature and salt concentration of the solution. The thermal denaturation temperature and enthalpy of the extracted GPC also differed among the two protein extraction methods-acid precipitation and enzymatic extraction. The in vitro protein digestibility of the GPC, assessed in terms of free amino N release during digestion, was observed to be quite low during the gastric digestion phase but increased during small-intestinal digestion phase. This was possibly related to the increase of the solubility of the GPC during the latter phase of digestion. Changes in protein solubility of GPC dispersions through the use of surfactants will be studied in the future as these amphiphilic molecules tend to bind to proteins, shielding their hydrophobic regions, thereby increasing solubility. The use of innovative technologies, including the use of ultrasound and pulsed electric fields, alone and in combination with the traditional extraction methods could be explored in the future to improve the yield and functional characteristics of the protein concentrates from grass.

## Figures and Tables

**Table 1:** Proximate analysis of GPC extracted through acid precipitation.

Component	Proportion (% or mg/100 g)
Major components (%)	
Moisture	3.77%
Ash	2.53%
Protein	54.69%
Fat	8.45%
Carbohydrate	26.61%
Insoluble Dietary Fibre	18.40%
Soluble Dietary Fibre	3.60%
Minerals (mg/100 g)	
Calcium	143 mg/100 g
Magnesium	95 mg/100 g
Potassium	320 mg/100 g
Sodium	121 mg/100 g
Iron	40 mg/100 g

## Data Availability

The data are available on request from the corresponding author, (J.S.).
